# From Plant to Target: Uncovering a Novel Anti-Inflammatory Compound in *Pouzolzia pentandra* via Phytochemical, Cellular, and Computational Studies

**DOI:** 10.3390/molecules31030461

**Published:** 2026-01-28

**Authors:** Do Tien Lam, Nguyen Anh Hung, Dao Viet Hung, Pham Thi Hong Minh, Hoang Thi Le Thuong, Vu Thi Thu Le

**Affiliations:** 1 Institute of Chemistry, Vietnam Academy of Science and Technology, Nghia Do, Hanoi City 100000, Vietnam; 2 Faculty of Chemistry, Hanoi Pedagogical University 2, Vinh Phuc, Phuc Yen 280000, Vietnam; 3 Department of Basic Science, Thai Nguyen University of Agriculture and Forestry (TNU), Quyet Thang, Thai Nguyen 24119, Vietnam; 4 Faculty of Education, Tan Trao University, Tuyen Quang, Tuyen Quang 02073, Vietnam

**Keywords:** molecular dynamics, docking, pouzolignan F, cyclooxygenase-2, phosphodiesterase-4

## Abstract

Phytochemical investigation of the ethyl acetate extract from the aerial parts of *Pouzolzia pentandra* led to the isolation and identification of fourteen compounds (**1**–**14**). These include known compounds such as *β*-sitosterol (**1**), bauerenol (**2**), oleanolic acid (**3**), 3*β*-friedelanol (**4**), kaempferol (**5**), quercetin (**6**), 2′,6′-dihydroxy-3′,4′-dimethoxychalcone (**7**), friedelan-3-one (**8**), dipterocarpol (**9**), 3*β*-hydroxyolean-12-en-28-one 3-*p*-coumarate (**10**), daucosterol (**11**), astilbin (**12**), 3-methoxy-4-hydroxybenzoic acid (**13**), and pouzolignan F (**14**). Among these, compound **14** displayed the most potent inhibitory activity on nitric oxide (NO) production in LPS-stimulated RAW264.7 macrophages, with an IC_50_ value of 10.54 ± 0.4 µM. Mechanistic studies further revealed that compound **14** significantly suppressed the LPS-induced release of key pro-inflammatory cytokines, tumor necrosis factor-alpha (TNF-*α*) and interleukin-6 (IL-6). Furthermore, it inhibited the activation of the nuclear factor-kappa B (NF-*κ*B) signaling pathway by preventing the nuclear translocation of its p65 subunit. Molecular docking studies were performed to evaluate the anti-inflammatory potential of compound **14** against cyclooxygenase-2 (COX-2) and phosphodiesterase-4 (PDE4). The compound exhibited binding affinities of −6.138 kcal/mol and −9.361 kcal/mol for COX-2 and PDE4, respectively. Subsequent molecular dynamics (MD) simulations confirmed the formation of a stable complex with the active site of PDE4. Collectively, these integrated in vitro and in silico findings demonstrate that pouzolignan F acts as a multi-target anti-inflammatory agent, likely through the inhibition of inflammatory mediators, cytokines, and the NF-*κ*B pathway.

## 1. Introduction

The genus *Pouzolzia* (Urticaceae), distributed throughout tropical and subtropical regions, comprises over 50 species globally, with approximately six documented in Vietnam [1,2,3]. Plants in this genus are frequently used in traditional medicine to treat various ailments [3,4,5]. Specifically, *P. pentandra* is used for conditions such as dermatitis, urinary disorders, menstrual issues, and periodontitis [3]. These traditional applications suggest the presence of bioactive constituents, which have been preliminarily identified to include flavonoids, anthraquinones, alkaloids, and terpenoids [5,6,7].

Investigations into the biological activities of *P. pentandra* have reported several effects. Extracts from its stems and leaves have shown antibacterial activity against common pathogens like *Staphylococcus aureus* and *Escherichia coli* [7,8,9]. Furthermore, a methanol leaf extract demonstrated antiproliferative activity against human hepatocellular carcinoma HepG2 cells, with a reported IC_50_ value of 35.51 μg/mL [10]. The plant’s antioxidant capacity has been more extensively documented. Its ethanol leaf extract exhibits radical scavenging activity against 2,2-diphenyl-1-picrylhydrazyl (DPPH) (IC_50_ = 43.2 ± 0.4 μg/mL) and inhibits lipid peroxidation (IC_50_ = 37.1 ± 0.6 μg/mL). These activities correlate with a total phenolic content of 14.4 ± 1.2 mg gallic acid equivalents per gram (mg GAE/g) [11,12]. A separate study on a 95% ethanol extract reported even more potent antioxidant activity across multiple assays, including DPPH radical scavenging (DPPH EC_50_ = 17.29 ± 0.26 μg/mL) and 2,2′-azino-bis(3-ethylbenzothiazoline-6-sulfonic acid) radical cation scavenging (ABTS EC_50_ = 9.92 ± 0.29 μg/mL) [6].

In contrast to the antioxidant and antimicrobial properties, the anti-inflammatory potential of *P. pentandra* remains less rigorously established. While not directly assessed in detail, its potential can be inferred from several lines of evidence. First, known anti-inflammatory compounds such as quercetin and kaempferol have been isolated from this species [10,13,14,15]. Second, congeneric species like *P. zeylanica* and *P. sanguinea* have demonstrated notable anti-inflammatory effects in vitro, often measured by the inhibition of NO production in LPS-stimulated macrophages [16,17,18,19,20,21,22]. Finally, the strong antioxidant activity of *P. pentandra* is relevant due to the intrinsic link between oxidative stress and inflammatory pathways [23,24,25,26,27,28,29]. Despite these indications, a comprehensive investigation specifically targeting the anti-inflammatory mechanisms of *P. pentandra* is lacking.

To bridge this knowledge gap, a multi-faceted research approach is warranted. Modern drug discovery often leverages in silico virtual screening to efficiently identify potential bioactive compounds from complex mixtures by predicting their binding affinity to key therapeutic targets [30,31,32,33,34,35,36,37]. For inflammation, COX-2 and PDE4 are critical enzymes regulating the inflammatory response [38,39,40,41,42,43]. This computational method serves as a valuable preliminary step to prioritize compounds for further experimental validation.

Therefore, this study aims to systematically evaluate the anti-inflammatory potential of *P. pentandra* through an integrated approach. We will employ in silico virtual screening to identify compounds within an extract library that exhibit strong binding affinity to COX-2 and PDE4. The most promising candidates will then be experimentally validated using in vitro assays. This work seeks to provide a clearer scientific foundation for the traditional uses of *P. pentandra* and contribute to the discovery of novel anti-inflammatory agents from natural sources.

## 2. Results and Discussions

### 2.1. Phytochemical Characterization of P. pentandra

**Figure 1:**
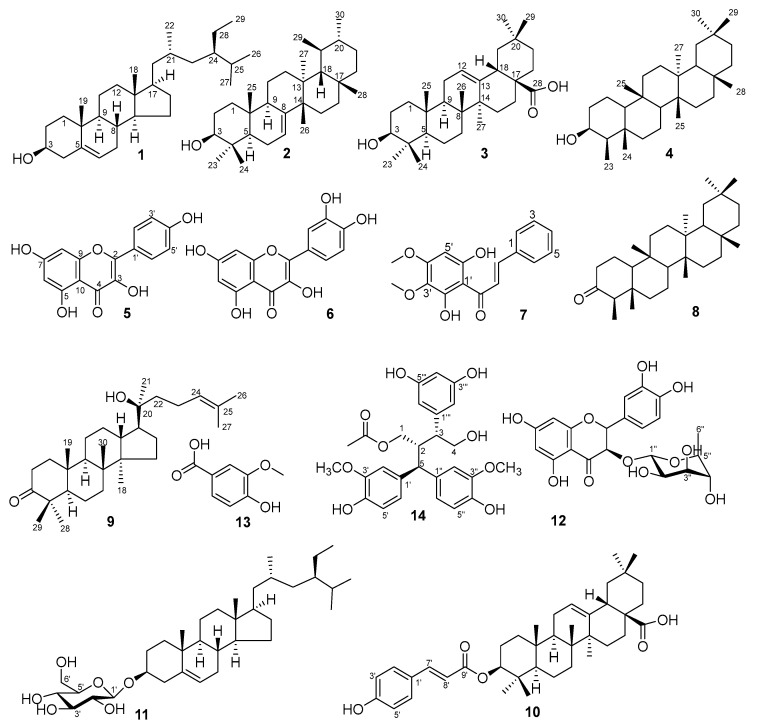
Chemical structures of isolated compound from *P. pentandra*.

Phytochemical investigation of the ethyl acetate extract of *P. pentandra* led to the isolation of fourteen known compounds (**1**–**14**). The compounds represent diverse classes: sterols (**1**, **11**), triterpenoids (**2**–**4**, **8**–**10**), flavonoids (**5**, **6**, **12**), a chalcone (**7**), a phenolic acid (**13**), and a lignan (**14**) (Figure 1). This profile aligns with the known chemistry of the *Pouzolzia* genus [3,6,10]. Notably, compounds **7**, **10**, and **14** are reported for the first time from *P. pentandra*. The presence of known anti-inflammatory agents like kaempferol (**5**) and quercetin (**6**) provides a chemical basis for the plant’s traditional use [13].

Compound **1** was identified as *β*-sitosterol, characterized by NMR signals for a hydroxyl-bearing methine [δ_H_ 3.53 (δ_C_ 71.7)] and an olefinic proton [δ_H_ 5.35 (δ_C_ 140.7, 121.7)] [44]. Compound **2**, a colorless solid, was identified as bauerenol (ursane-type triterpenoid). Key NMR data included eight methyl signals (δ_H_ 0.75–1.03), a hydroxyl-bearing methine (δ_H_ 3.20, H-3), and thirty carbon signals consistent with the structure [45]. Compound **3** was characterized as oleanolic acid. Diagnostic signals included a carboxyl carbon (δ_C_ 183.0), olefinic protons/carbons [δ_H_ 5.29 (H-12); δ_C_ 122.7, 143.6], a hydroxymethine [δ_H_ 3.23 (H-3*β*); δ_C_ 79.0], and seven methyl singlets [46]. Compound **4** exhibited characteristic friedelane-type signals: eight methyls (seven singlets, one doublet at δ_H_ 0.93) and a hydroxymethine (δ_H_ 3.71, H-3; δ_C_ 72.8). The data matched 3*β*-friedelanol [47]. Compound **5** displayed characteristic kaempferol NMR signals: meta-coupled A-ring protons [δ_H_ 6.44 (H-8), 6.19 (H-6)], *ortho*-coupled B-ring protons [δ_H_ 8.05 (H-2′/6′), 6.93 (H-3′/5′)], and a carbonyl (δ_C_ 175.9, C-4) [48]. Compound **6** was identified as quercetin, with aromatic protons [δ_H_ 7.67 (H-2′), 7.54 (H-6′), 6.87 (H-5′), 6.40 (H-8), 6.18 (H-6)], multiple hydroxyls, and a carbonyl (δ_C_ 175.8) [49].

Compound **7** was determined to be 2′,6′-dihydroxy-3′,4′-dimethoxychalcone. Key features included trans-olefinic protons [δ_H_ 7.94 (H-*β*), 7.75 (H-*α*)], A-ring protons (δ_H_ 7.66–7.41), B-ring signals [δ_H_ 6.10 (H-5′), 3.92/3.80 (OCH_3_)], and a carbonyl (δ_C_ 194.4) [50]. Compound **8** was identified as friedelan-3-one. The ^1^H-NMR spectrum showed seven methyl singlets and one methyl doublet, while the ^13^C-NMR revealed a characteristic ketone at C-3 (δ_C_ 213.2) [51]. Compound **9** exhibited sterol signals including an olefinic proton [δ_H_ 5.12 (H-24)] and a carbonyl (δ_C_ 218.1, C-3), identifying it as dipterocarpol [52]. Compound **10** was characterized as 3*β*-hydroxyolean-12-en-28-one 3-*p*-coumarate. NMR data showed oleanane-type signals [δ_H_ 5.19 (H-12), 4.63 (H-3)] coupled with a cinnamoyl moiety and an ester carbonyl (δ_C_ 165.7) [53]. Compound **11** was identified as daucosterol, evidenced by an olefinic proton (δ_H_ 5.30), a glycosidic doublet [δ_H_ 4.32 (*J* = 7.8 Hz); δ_C_ 100.9], and seven oxygenated carbons [54]. Compound **12** was established as astilbin, a flavonoid glycoside. Key data included a carbonyl (δ_C_ 196.0), flavonoid protons (δ_H_ 5.11, 4.61), and a rhamnopyranoside moiety [55]. Compound **13** was identified as 3-methoxy-4-hydroxybenzoic acid, showing aromatic protons [δ_H_ 7.58 (H-6), 7.57 (H-2), 6.84 (H-5)] and a methoxy group (δ_H_ 3.91) [56]. Compound **14** was conclusively identified as pouzolignan F based on NMR signals for nine aromatic protons, two hydroxymethylenes, two methoxyls, one methyl, and a carbonyl (δ_C_ 172.8) [4,22].

It is noteworthy that several intermediate fractions obtained during the chromatographic separation (e.g., **PPE2.1**, **PPE2.2**, **PPE3.2**) were not pursued for full isolation in this study due to their complex composition or minute quantities of constituents. These fractions represent a reservoir of unexplored chemical diversity within *P. pentandra* and warrant further investigation in future studies aimed at discovering minor or novel bioactive compounds.

### 2.2. Inhibition of Nitric Oxide Production in LPS-Stimulated Macrophages

NO is a key inflammatory mediator produced by inducible nitric oxide synthase (iNOS) in activated macrophages. Excessive NO production is implicated in the pathogenesis of chronic inflammatory diseases. Therefore, the inhibition of NO release is a standard assay for evaluating anti-inflammatory potential [57,58,59,60,61,62,63,64,65,66,67].

**Figure 2:**
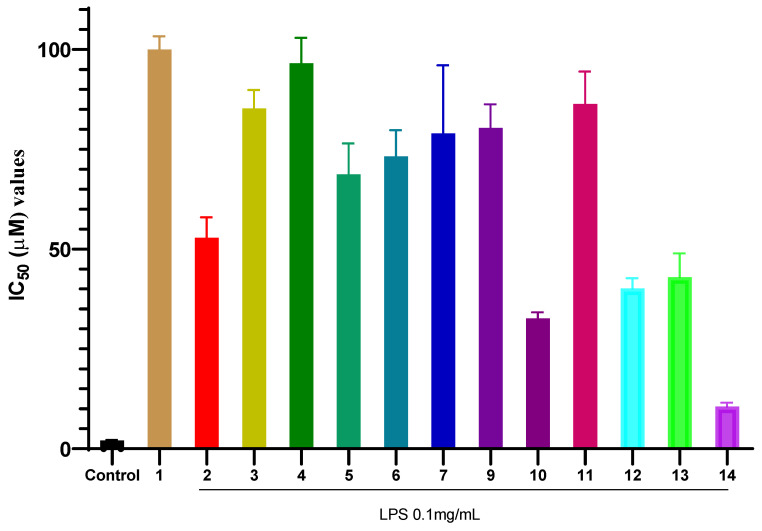
Inhibitory effects of compounds **1**–**14** on NO production in LPS-stimulated RAW 264.7 macrophages. Values represent the mean IC_50_ ± SD from three independent experiments (n = 3). Control (+) is cardamonin.

The inhibitory effects of the isolated compounds (**1**–**14**) on NO production in LPS-stimulated RAW 264.7 macrophages are summarized in Figure 2. Among all tested compounds, compound **14** exhibited the most potent activity with an IC_50_ value of 10.54 ± 0.40 µM. The IC_50_ value of compound **14** was approximately five times higher (indicating lower potency) than that of the positive control, cardamonin (IC_50_ = 2.08 ± 0.05 µM), a known natural anti-inflammatory chalcone used as a reference in this assay. Cardamonin, a natural chalcone, is a well-established reference compound in anti-inflammatory research, known for its potent inhibition of NO production primarily through suppression of the NF-*κ*B and MAPK signaling pathways [57]. Compound **10** and compound **12** showed moderate inhibitory effects, with IC_50_ values of 32.65 ± 0.60 µM and 40.16 ± 1.03 µM, respectively. In contrast, common phytoconstituents such as compounds **1**, **3**, and **6** demonstrated weak inhibitory activity, with IC_50_ values exceeding 70 µM. These results clearly highlight compound **14** as the most promising anti-inflammatory candidate isolated from *P. pentandra* in this study, warranting further mechanistic investigation.

The marked potency of compound **14** highlights the significance of the lignan skeleton in mediating anti-inflammatory effects. Lignans from other plant sources, such as *Schisandra chinensis*, have been widely reported for their anti-inflammatory properties, often through the modulation of NF-*κ*B and MAPK pathways [55]. The superior activity of **14** compared to flavonoids and simple triterpenoids in our assay suggests its unique mechanism may involve multiple targets.

The enhanced activity of the triterpenoid-coumarate ester (**10**) over its aglycone oleanolic acid (**3**) is noteworthy. Esterification with p-coumaric acid likely increases lipophilicity and membrane permeability and introduces additional antioxidant pharmacophores (phenolic hydroxyls). This aligns with studies showing that esterification or glycosylation of triterpenoid acids can significantly augment their anti-inflammatory potency by improving bioavailability and target engagement [53].

The moderate activity of compound **12**, a dihydroflavonol glycoside, compared to the weak activity of the aglycones quercetin and kaempferol, suggests that glycosylation may influence cellular uptake, stability, or specific target interaction in the context of NO inhibition. This is consistent with some studies where flavonoid glycosides showed different or sometimes enhanced bioactivity compared to their aglycones due to altered pharmacokinetics [55].

**Figure 3:**
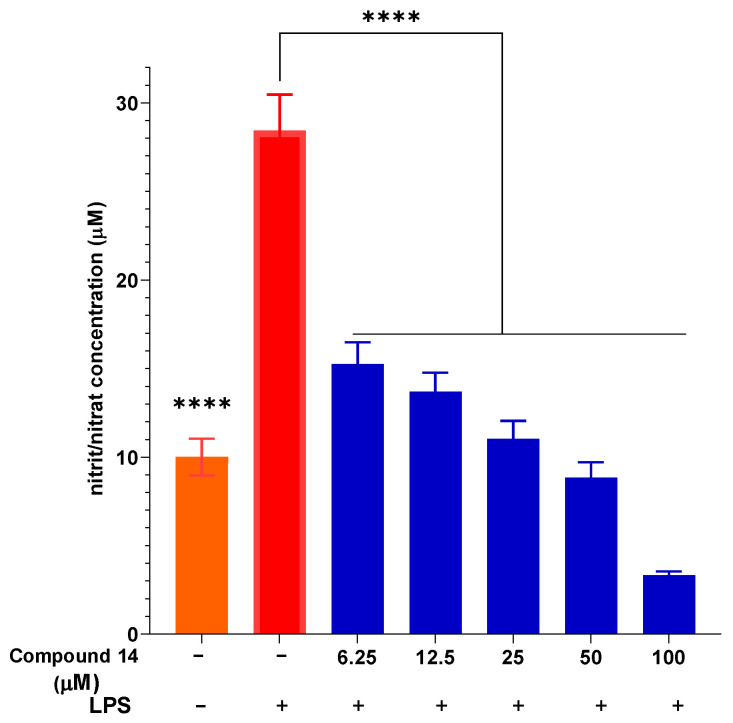
The ability of compound **14** to inhibit intracellular NO production with LPS stimulation. Data are presented as mean ± SD of three independent experiments, each performed in triplicate (n = 3). ****: *p* < 0.0001 compared to the treatment with only the presence of LPS.

The concentration-dependent inhibition of NO production by compound **14** was further investigated (Figure 3). Upon LPS stimulation, NO concentration increased significantly to 28.43 ± 2.05 µM compared to the unstimulated control. Pre-treatment with compound **14** resulted in a dose-dependent suppression of NO release. At the lowest concentration tested (6.25 µM), NO levels were reduced to 15.26 ± 1.23 µM, representing approximately 46% inhibition. This inhibitory effect became more pronounced at higher concentrations, reaching 88% inhibition at 100 µM (3.34 ± 0.21 µM). These results not only confirm the potent activity of compound **14** but also demonstrate its consistent dose–response behavior, which is a key pharmacological characteristic for a potential lead compound.

**Figure 4:**
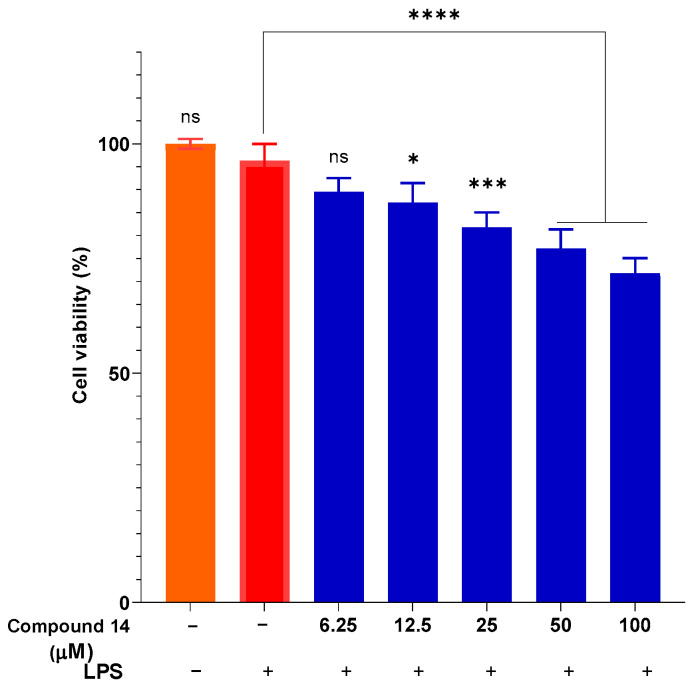
Cytotoxicity of compound **14** in the presence of LPS. Data are presented as mean ± SD of three independent experiments, each performed in triplicate (n = 3). ****: *p* < 0.0001, ***: *p* < 0.001, and *: *p* < 0.05 compared to cells cultured under normal conditions; n.s.: not significant.

To ensure that the observed anti-inflammatory effects were not due to cytotoxicity, cell viability was assessed under the same treatment conditions (Figure 4). The results indicate that compound **14** did not exhibit significant cytotoxicity at concentrations up to 50 µM, with viability maintained above 77%. Even at the highest concentration tested (100 µM), cell viability remained above 70%, which is generally considered acceptable for in vitro anti-inflammatory screening. This confirms that the inhibition of NO production by compound **14** is indeed due to its specific anti-inflammatory activity and not a result of reduced cell viability.

### 2.3. Suppression of Pro-Inflammatory Cytokines (TNF-α and IL-6)

**Figure 5:**
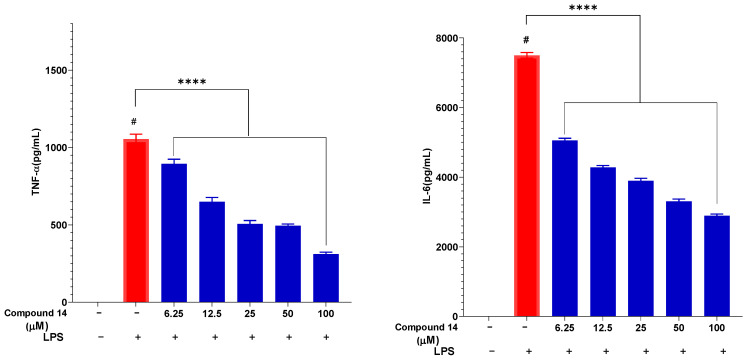
Inhibitory effect of compound **14** on the secretion of pro-inflammatory cytokines: TNF-α and IL-6. Data are expressed as mean ± SD from three independent experiments (n = 3). ****: *p* < 0.0001 compared to the treatment with only the presence of LPS. #: *p* < 0.05 compared to cells cultured under normal conditions.

The effect of compound **14** on the secretion of key pro-inflammatory cytokines, TNF-α and IL-6, was also evaluated in a dose-dependent manner (Figure 5). LPS stimulation markedly increased TNF-α and IL-6 levels to 1055 ± 30.65 pg/mL and 7500 ± 80.34 pg/mL, respectively. Pre-treatment with compound **14** significantly reduced the release of both cytokines in a concentration-dependent manner. At 100 µM, TNF-α and IL-6 levels were suppressed to 311 ± 12.05 pg/mL and 2895 ± 45.21 pg/mL, corresponding to inhibition rates of approximately 70% and 61%, respectively. This dual cytokine suppression suggests that compound **14** acts on upstream regulators of the inflammatory cascade, potentially through modulation of shared signaling pathways such as NF-*κ*B.

Building on its NO inhibitory activity, compound **14** was further evaluated for its effect on cytokine secretion. Pre-treatment with compound **14** significantly and dose-dependently suppressed the LPS-induced release of both TNF-α and IL-6 in RAW 264.7 cells (Figure 5). This dual inhibition is of considerable pharmacological importance. TNF-α is a master upstream regulator that amplifies inflammation and induces the production of other cytokines, including IL-6. IL-6 itself is a pivotal mediator in acute and chronic inflammation. The ability of compound **14** to simultaneously downregulate these key cytokines suggests it intervenes at a central node in the inflammatory signaling network, potentially preventing the cascade’s propagation. This multi-cytokine inhibitory profile is a desirable feature shared by some biologic drugs and potent natural products like curcumin.

### 2.4. Inhibition of the NF-κB Signaling Pathway

The NF-*κ*B pathway is a primary regulator of inflammatory gene expression, controlling the transcription of iNOS, TNF-α, IL-6, COX-2, and other mediators.

**Figure 6:**
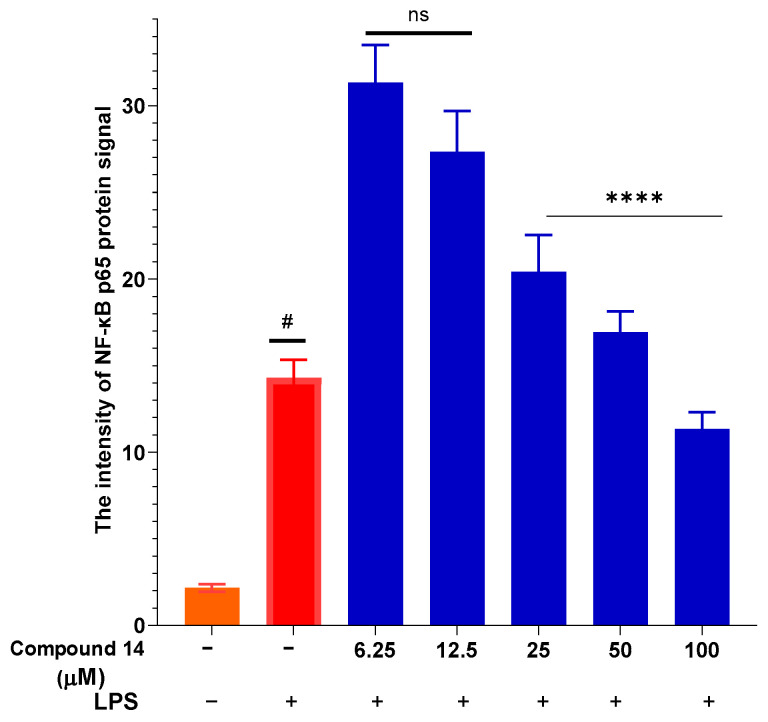
Inhibition of NF-*κ*B p65 nuclear translocation by compound **14**: Quantification of nuclear p65 fluorescence intensity. Data points represent individual cells from three independent experiments (total n = 36 cells). Values are mean ± SEM. #: *p* < 0.05 (compared to cells cultured under normal conditions); ****: *p* < 0.0001 (compared to cells stimulated with LPS). n.s.: no significant difference.

To elucidate the mechanism underlying the suppression of NO and cytokines, the effect of compound **14** on NF-*κ*B activation was assessed by measuring nuclear translocation of the p65 subunit (Figure 6). LPS stimulation led to a significant increase in nuclear p65 intensity, indicative of pathway activation. Treatment with compound **14** resulted in a dose-dependent reduction in p65 signal intensity, with the most pronounced inhibition observed at 100 µM. This indicates that 14 effectively blocks NF-*κ*B activation, providing a mechanistic explanation for its downregulation of downstream inflammatory mediators such as iNOS, TNF-α, and IL-6.

Our results position compound **14** as a multi-target anti-inflammatory agent acting, at least in part, through the suppression of the NF-*κ*B pathway. This mechanism is shared by many classic anti-inflammatory drugs (e.g., corticosteroids) and numerous plant-derived compounds. The novelty here lies in identifying this specific lignan from *P. pentandra* as a potent NF-*κ*B inhibitor. Compared to other anti-inflammatory lignans reported in the genus, such as those from *P. sanguinea* [4,22], compound **14** appears to exhibit comparable or superior activity in NO inhibition assays, warranting further comparative studies.

The inhibition of NF-*κ*B is intrinsically linked to antioxidant activity, as reactive oxygen species (ROS) are key activators of this pathway [23,24]. While not measured in this study, the antioxidant potential of compound **14**, inferred from its phenolic structure, might contribute to its anti-inflammatory effect by scavenging ROS and thus indirectly suppressing NF-*κ*B activation. This forms a logical connection to the reported antioxidant activities of *P. pentandra* extracts [6,11].

### 2.5. In Silico Molecular Docking

To complement the in vitro findings and explore potential direct enzyme targets, computational studies were performed.

**Figure 7:**
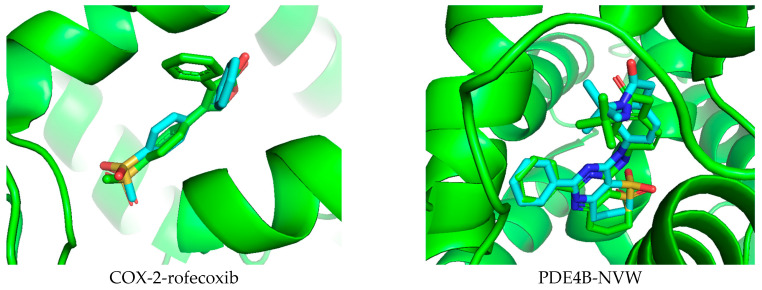
Validation of the docking protocol by extracting and re-docking the co-crystallized ligand in the active site of the studied protein.

**Figure 8:**
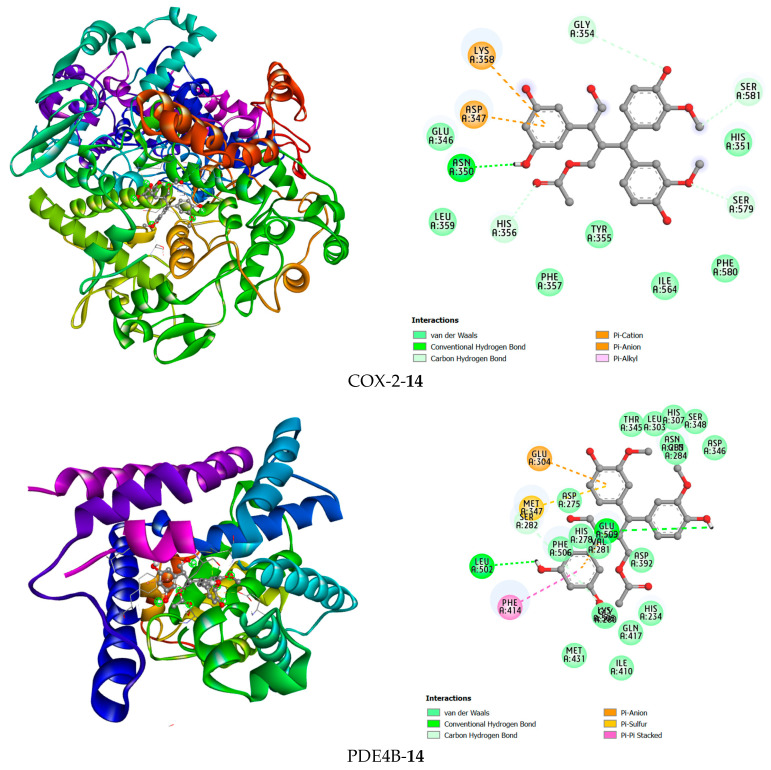
The 3D and 2D interaction views of compound **14** with COX-2 and PDE4B.

Docking studies were conducted in this research to better understand the potential anti-inflammatory capabilities of compound **14** against known targets, including the enzymes COX-2 and PDE4B. Before evaluation, the docking protocol was validated by redocking the co-crystallized ligands into the active sites of the studied enzymes. The results described in Figure 7 show that the calculated RMSD values were 1.672 Å and 0.615 Å for rofecoxib in the COX-2 protein and NVW in the PDE4B protein, respectively. RMSD values below 2 Å indicate that the docking protocol has high predictive reliability and was thus used in the current study [62]. Subsequently, compound **14** was docked into the active sites of the studied enzymes. The best binding positions of the ligand with COX-2 and PDE4B are shown in Figure 7, indicating their interactions with the amino acid residues of these proteins. The binding affinities of compound **14** and the reference compounds were also calculated, providing a basis for assessing how these ligands bind to the target proteins COX-2 and PDE4B, as shown in Table 1. It can be seen that compound **14** exhibits predicted binding affinity values of −6.138 kcal/mol and −9.361 kcal/mol for the COX-2 and PDE4B proteins, respectively. The value for PDE4B indicates a strong predicted affinity in silico. However, compared to the high-affinity reference inhibitors rofecoxib (COX-2: −8.9 kcal/mol) and NVW (PDE4B: −12.05 kcal/mol), compound **14** shows weaker predicted binding for both targets. Notably, the docking pose for PDE4B reveals interactions with key residues like Phe414 in the hydrophobic clamp, a feature critical for known PDE4 inhibitors, which may explain its significant activity in subsequent cellular assays despite the lower predicted affinity score relative to NVW. A binding interaction analysis was conducted and is depicted in Figure 8.

Compound **14** forms hydrogen bonds in the active site of the PDE4B enzyme with the two amino acid residues Leu502 and Glu509. Additionally, hydrophobic interactions were found between this ligand and Glu304 and Glu509 in a pi-anion interaction manner, Met347 in a pi-sulfur interaction manner, and Phe414 in a pi-pi stacked interaction manner. In the COX-2 active site, compound **14** was observed to form a hydrogen bond with Asn350. Furthermore, this compound has hydrophobic interactions with the amino acid residues Lys358, Asp347, His356, Ser581, Ser579, and Gly354 (Figure 8).

For PDE4B, the high-affinity inhibitor NVW extensively interacts with the metal ion-binding pocket and the hydrophobic clamp (e.g., Phe414). Impressively, compound **14** managed to form hydrogen bonds with Leu502 and Glu509, and also engaged Phe414 via a pi-pi stacked interaction, mirroring a key interaction observed with NVW. This ability to target the conserved hydrophobic clamp residue Phe414, which is critical for inhibitor binding in PDE4, provides a strong rationale for its significant inhibitory activity observed in biochemical assays (Table 1).

The stronger predicted affinity for PDE4B over COX-2 is intriguing. PDE4 inhibitors are a recognized class of anti-inflammatory agents used in conditions like psoriasis and COPD [41,42]. The predicted interaction of 14 with PDE4B, particularly with Phe414, suggests it might function as a novel natural PDE4 inhibitor scaffold. This is a significant finding as most reported PDE4 inhibitors from plants are flavonoids (e.g., from *Millettia dielsiana* [62]); a lignan with this activity is less common.

An important consideration is that our docking protocol was targeted at the canonical active sites. While the results indicate a potential orthosteric interaction, the possibility of compound **14** binding to an allosteric site on COX-2 or PDE4B was not investigated in this study. This represents a limitation of our current computational approach and an interesting direction for future work

### 2.6. Molecular Dynamics

**Figure 9:**
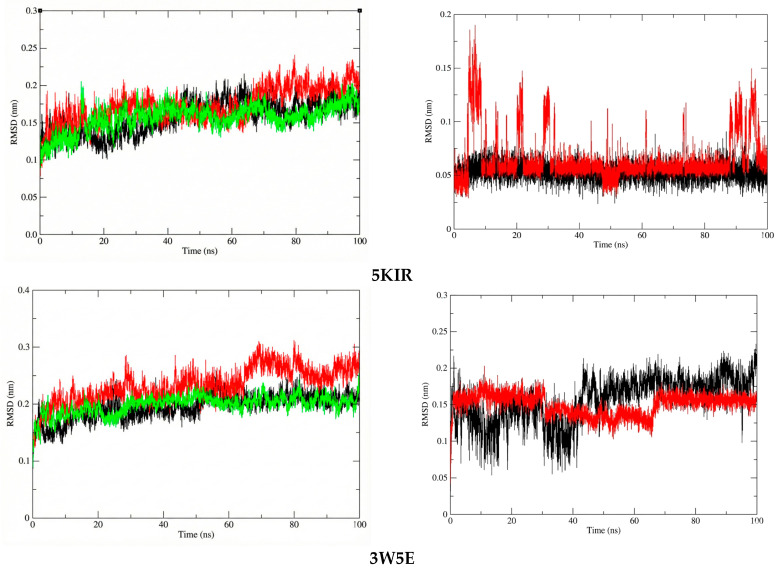
The RMSD plots of protein in systems (**left**) and ligand in systems (**right**). The systems are color-coded as apo (black), reference (red), and ligand **14** (green).

To assess the structural stability and compactness of the protein-ligand complexes obtained from molecular docking simulations, a MD approach was employed in this study. First, we calculated the differences in the structural stability of the backbone atoms and ligands in the **14**-5KIR, **14**-3W5E, rofecoxib-5KIR, and NVW-3W5E systems by computing the RMSD values. The RMSD values of the investigated complexes were compared with those of the reference complexes, as presented in Figure 9. Analysis of the RMSD indicated that the protein structures in complexes with compound **14** (COX-2-14 and PDE4B-14) attained a higher degree of conformational stability compared to the reference inhibitor complexes, maintaining lower and more consistent RMSD values during the 100 ns MD simulation. Specifically, the average RMSD values for the COX2-14, PDE4B-14, COX2-rofecoxib, PDE4B-NVW, apo-COX-2, and apo-PDE4B systems were 0.156784267 nm, 0.1986632 nm, 0.173008612 nm, 0.232984298 nm, 0.158096786 nm, and 0.195830385 nm, respectively.

At the molecular biology level, compound **14** formed a highly stable complex with the active site of PDE4 compared to COX-2. This stability is attributed to the absence of significant structural changes in the ligand within the PDE4B complex, whereas in the active site of COX-2, there were periods of structural changes, and the RMSD values continued to fluctuate even at the end of the simulation. However, throughout the entire simulation, the RMSD values of compound **14** in both investigated systems remained below 0.2 nm (2 Å), indicating minimal structural changes. Another noteworthy point is that compound **14** in the PDE4B complex had a lower average RMSD value compared to the reference compound NVW, with values of 0.150194 nm and 0.156813 nm, respectively.

**Figure 10:**
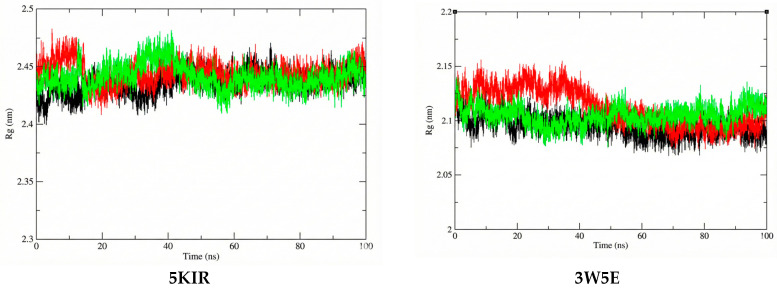
The Rg plots of the studied complexes. The systems are color-coded as apo (black), reference (red), and ligand **14** (green).

The Rg was estimated in this study to reflect the compactness of the investigated complexes. The average Rg values calculated for the **14**-5KIR, **14**-3W5E, rofecoxib-5KIR, and NVW-3W5E complexes were 2.441395 nm, 2.104632964 nm, 2.444086 nm, and 2.111742684 nm, respectively (Figure 10). There were no structural changes in the acetylcholinesterase configuration in the presence of the ligands, and a stable equilibrium radius of gyration was established, implying the stability of the complexes during the 100 ns simulation. The calculated Rg values of the studied systems are shown in Figure 10. This high structural stability suggests that researchers should experimentally test these compounds in vitro and in vivo to elucidate their anti-inflammatory mechanisms.

### 2.7. Integrated Discussion and Implications

This study successfully bridges ethnopharmacology, phytochemistry, and molecular pharmacology to validate the anti-inflammatory potential of *P. pentandra*. We have identified compound **14** as its most active anti-inflammatory constituent to date.

The multi-faceted activity profile of compound **14**—inhibiting NO, TNF-α, IL-6, and NF-*κ*B activation is highly desirable. It suggests a mechanism that is both broad (affecting multiple mediators) and upstream (targeting central transcription). This could translate to efficacy in complex inflammatory conditions where multiple pathways are dysregulated.

The integrated in silico and in vitro approach provides a robust hypothesis for its action: the potent cellular activity is likely driven primarily by inhibition of the NF-*κ*B pathway, while direct, moderate inhibition of enzymes like PDE4B may contribute synergistically. This multi-target action aligns with the network pharmacology concept often observed for effective natural products.

Previous studies on *P. pentandra* focused on its antioxidant and antimicrobial properties [8,9,10,11,12], with only indirect evidence for anti-inflammatory activity. Studies on related species (*P. zeylanica*, *P. sanguinea*) reported anti-inflammatory effects of extracts and some isolated flavonoids or norlignans [16,17,18,19,20,21,22]. Our work is the first comprehensive report isolating a broad range of compounds from *P. pentandra* and systematically evaluating their NO inhibitory activity, leading to the identification of a potent lignan lead. Furthermore, we provide the first mechanistic insight for any compound from this species, demonstrating NF-*κ*B pathway inhibition. The computational prediction of PDE4B targeting also opens a new avenue for understanding its mode of action.

The potent activity of compound **14** prompts consideration of its potential for optimization through chemical derivatization. While beyond the scope of this discovery study, future work could explore modifying its key pharmacophoric features—such as the phenolic hydroxyls, carbonyl group, and methoxy substitution pattern—to potentially enhance potency, improve pharmacokinetic properties, or refine its multi-target activity profile.

This study has limitations. The in vitro models, while standard, do not fully replicate in vivo complexity. The direct inhibition of COX-2, PDE4, or other enzymes by compound **14** needs in vitro enzymatic assay validation. Furthermore, the pharmacokinetic properties and in vivo efficacy of compound **14** in animal models of inflammation (e.g., carrageenan-induced paw edema, DSS-induced colitis) are crucial next steps to assess its therapeutic potential.

Moreover, to experimentally verify the docking-predicted interaction with COX-2 and determine its mode of action, future work should include in vitro enzyme inhibition assays. These assays would involve testing compound **14** in the presence of a known competitive inhibitor like rofecoxib to conclusively establish whether it acts as a competitive inhibitor.

Furthermore, while this study establishes the in vitro potency of compound **14**, its therapeutic potential relative to clinical agents requires careful interpretation. The IC_50_ value against NO production (~10 µM) places it within an active range observed for some natural product leads. A direct comparison with the in vitro potencies of classic NSAIDs (e.g., indomethacin, celecoxib) is challenging due to differences in primary molecular targets, assay conditions, and the fact that clinical efficacy stems from a complex interplay of pharmacokinetic and pharmacodynamic properties [63,64]. Therefore, the significant activity demonstrated here, coupled with its multi-target mechanistic profile (NF-*κ*B, potential PDE4 interaction), strongly justifies the recommended next step: comprehensive in vivo evaluation in established animal models of inflammation to truly assess its translational relevance and therapeutic promise.

Taken together, the data from Figure 2, Figure 3, Figure 4, Figure 5 and Figure 6 demonstrate that compound **14** is a multi-target anti-inflammatory agent with a promising pharmacological profile. It potently inhibits NO production, suppresses pro-inflammatory cytokines (TNF-α and IL-6), and attenuates NF-*κ*B activation-all at non-cytotoxic concentrations. These findings not only validate the traditional use of *P. pentandra* in inflammatory conditions but also highlight compound **14** as a novel lignan lead worthy of further in vivo and mechanistic studies.

## 3. Materials, Methods and Experiment

### 3.1. Plant Materials

The samples were identified by Dr. Nguyen Quoc Binh, Vietnam National Museum of Nature (VAST) under the scientific name as *P. pentandra* (Roxb.) Benn., Urticaceae family. A voucher specimen was deposited at Hanoi Pedagogical University 2, Phuc Yen, Vinh Phuc, Vietnam.

### 3.2. Methods of Separation, Isolation and Structure Determination of Compounds

Thin layer chromatography (TLC) was performed on a DC-Alufolien 60 F254 pre-coated silica gel plate (Merck, Darmstadt, Germany) with a silica gel layer thickness of 0.2 mm. Column chromatography (CC) was performed on silica gel (Kieselgel 60, 70–230 mesh and 230–400 mesh, Merck, Darmstadt, Germany) or RP-18 gel (30–50 μm, FuJi Silysia Chemical Ltd., Aichi, Japan). For TLC, pre-coated silica gel 60 F_254_ (0.25 mm, Merck, Darmstadt, Germany) and RP-18 F_254S_ (0.25 mm, Merck, Darmstadt, Germany) plates were used.

The structure of the isolated compounds were determined through the analysis of nuclear magnetic resonance spectra, one-dimensional spectroscopy techniques 1D–NMR (^1^H-NMR and ^13^C-NMR) and two-dimensional spectroscopy techniques 2D–NMR (HMBC and HSQC), performed on a Bruker Avance III 500 MHz spectrometer (Bruker Biospin, Faellanden, Switzerland) and comparison with data in the references.

### 3.3. Experiment and Separation

The aerial parts of the *P. pentandra* sample has been studied using the following standard procedure: The sample was cut into small pieces, air-dried, and then crushed to a coarse powder (yielding 3000 g). After that, the sample was extracted three times with methanol using an ultrasonic machine. The combined methanol extracts were concentrated under reduced pressure at a temperature below 50 °C to yield the crude methanol extract (**PP**, 110 g). The **PP** was mixed with a small amount of distilled water. This aqueous mixture was then subjected to liquid–liquid extraction with ethyl acetate (3 × 500 mL). The combined extracts were concentrated under reduced pressure using a rotary evaporator to obtain the ethyl acetate extract (**PPE**, 49 g) and the aqueous extract (**PPW**, 60 g), respectively.

The **PPE** (45 g) was roughly separated on a silica gel column chromatography using elution of *n*-hexane-ethyl acetate (HE 100:0 → 0:100) to yield six fractions (**PPE1–PPE6**).

The **PPE1** fraction (3.5 g) was separated on a silica gel column chromatography eluted with n-hexane-ethyl acetate (100:0 → 50:1) to yield three fractions (**PPE1.1–PPE1.3**). The **PPE1.1** and **PPE1.2** fractions were recrystallized in acetone to yield compounds **1** (*β*-sitosterol; 7.0 mg) and **2** (bauerenol; 8.5 mg), respectively.

The **PPE2** fraction (5.0 g) was separated on a silica gel column chromatography eluted with *n*-hexane-ethyl acetate (50:1 → 10:1) to obtain four small fractions (**PPE2.1–PPE2.4**). The **PPE2.3** fraction was recrystallized in acetone to yield compound **3** (oleanoic acid; 10.2 mg).

The **PPE3** fraction (6.8 g) was separated by RP18 column chromatography using elution of methanol–water (3:7 → 7:3) to produce five fractions (**PPE3.1–PPE3.5**). The **PPE3.2**, **PPE3.3** and **PPE3.4** fractions were separated further using Sephadex LH-20 column chromatography and eluting with methanol to give compounds **4** (3*β*-friedelanol; 8.0 mg), **5** (kaempferol; 9.3 mg) and **6** (quercetin; 6.1 mg), respectively.

The **PPE4** fraction (5.5 g) was separated by RP18 column chromatography using elution of methanol–water (3:7 → 7:3) to produce three fractions (**PPE4.1–PPE4.3**). The **PPE4.3** fraction was separated on a silica gel column chromatography eluted with dichloromethane–methanol (30:1 → 5:1) to obtain four small fractions (**PPE4.3.1–PPE4.3.4**). The **PPE4.2** fraction was recrystallized in acetone to yield compound **7** (2′,6′-dihydroxy-3′,4′-dimethoxychalcone; 5.9 mg) and **8** (friedelan-3-one; 11.5 mg), respectively.

The **PPE5** fraction (7.2 g) was separated by RP18 column chromatography using an elution of methanol–water (3:7 → 7:3) to produce three fractions (**PPE5.1–PPE5.3**). The **PPE5.2** fraction was separated on a silica gel column chromatography eluted with dichloromethane–methanol (20:1 → 2:1) to obtain three small fractions (**PPE5.2.1–PPE5.2.3**). The **PPE5.2.2** fraction was recrystallized in acetone to yield compound **9** (dipterocarpol; 4.3 mg). The **PPE5.1** and **PPE5.3** fractions were separated further using Sephadex LH-20 column chromatography and eluting with methanol to give compounds **10** (3*β*-hydroxyolean-12-en-28-one 3-p-coumarate; 7.2 mg) and **11** (daucosterol; 5.9 mg), respectively.

The **PPE6** fraction (3.4 g) was separated by RP18 column chromatography using elution of methanol–water (3:7 → 7:3) to produce five fractions (**PPE6.1–PPE6.5**). The **PPE6.2** and **PPE6.3** fractions were recrystallized in acetone to yield compounds **12** (astilbin; 5.6 mg) and **13** (3-methoxy, 4-hydroxy-benzoic acid; 8.9 mg). The **PPE6.4** fraction was separated further using Sephadex LH-20 column chromatography and eluting with methanol to give compound **14** (pouzolignan F; 12.5 mg). 

### 3.4. Inhibition of NO Production in LPS-Stimulated RAW 264.7 Macrophages

The inhibitory effect on the pro-inflammatory mediator NO was assessed in the murine macrophage cell line RAW 264.7. Cells were maintained in RPMI 1640 medium supplemented with 10% FBS, 100 U/mL penicillin, and 100 µg/mL streptomycin at 37 °C in a humidified 5% CO_2_ incubator. For the assay, cells were seeded at a density of 2 × 10^5^ cells/well into 96-well plates and allowed to adhere overnight. Cells were then pre-treated with various concentrations of the test samples or indomethacin (100 µg/mL, positive control) for 1 h, followed by stimulation with or without LPS (1 µg/mL) for 24 h. The cell-free supernatant was collected, and NO production was quantified by measuring the concentration of nitrite, a stable oxidative end product of NO, using the Griess reaction. Briefly, 100 µL of supernatant was mixed with an equal volume of Griess reagent (1% sulfanilamide, 0.1% N-1-naphthylethylenediamine dihydrochloride in 2.5% phosphoric acid) and incubated at room temperature for 10 min. The absorbance was measured at 540 nm. Sodium nitrite was used to generate a standard curve. The percentage inhibition of NO production was calculated relative to the LPS-stimulated control (no extract) [58,62,65].

### 3.5. Cell Viability Assay (MTT Assay)

To confirm that the observed inhibition of inflammatory mediators was not due to cytotoxicity, cell viability was assessed using the MTT assay on the same cells used for the NO production assay. Following supernatant collection for the Griess test, 20 µL of MTT solution (5 mg/mL in PBS) was added to each well containing the remaining cells and culture medium, resulting in a final concentration of 0.5 mg/mL. The plates were incubated at 37 °C for 3 h to allow formazan crystal formation. The supernatant was carefully removed, and 100 µL of DMSO was added to each well to dissolve the crystals. The plates were gently shaken for 10 min, and the absorbance was measured at 540 nm. Cell viability was expressed as a percentage relative to the untreated control cells (without LPS or test sample), using the formula: Viability (%) = (OD_test/OD_untreated control) × 100%. A test concentration was considered non-cytotoxic if it maintained cell viability at ≥80% [66,67].

### 3.6. Inhibition of Pro-Inflammatory Cytokine Secretion (TNF-α and IL-6)

The effect on the secretion of key pro-inflammatory cytokines was evaluated using enzyme-linked immunosorbent assay (ELISA). RAW 264.7 cells were seeded and treated following the same protocol as for the NO inhibition assay. After 24 h of co-incubation with test samples and LPS (1 µg/mL), the culture supernatants were collected and centrifuged to remove any cellular debris. The concentrations of secreted mouse TNF-α and IL-6 in the supernatants were quantified using commercial ELISA kits (Abcam) according to the manufacturer’s instructions. Briefly, standards and samples were added to antibody-precoated wells, followed by incubation with detection antibodies and substrate solution. The reaction was stopped, and the absorbance was measured at 450 nm with a reference wavelength of 570 nm. Cytokine concentrations were interpolated from the respective standard curves. The percentage inhibition of cytokine release was calculated relative to the LPS-stimulated control group [68,69].

### 3.7. Method for Assessing NF-κB Pathway Inhibition

The inhibitory effect of compound **14** on the NF-*κ*B signaling pathway was evaluated by examining the nuclear translocation of the p65 subunit using immunofluorescence staining. Procedure: RAW 264.7 macrophages were seeded onto chamber slides at a density of 2 × 10^6^ cells/well. Cells were pre-treated with the sample for 24 h, then stimulated with LPS (1 µg/mL) for 18 h. Subsequently, cells were fixed with 4% paraformaldehyde, permeabilized with 2% Triton-X, and blocked with 3% FBS containing 0.5% Tween 20. Samples were then incubated with an anti-NF-*κ*B p65 primary antibody, followed by a secondary antibody conjugated to Alexa Fluor 488. Cell nuclei were counterstained with DAPI. Images were captured using a fluorescent microscope (Olympus Fluoview FV101, Olympus Corporation, Tokyo, Japan), and the fluorescence intensity of p65 in the nuclei was quantified using Image J v1.54p and MetaMorph v7.8 software [70,71].

### 3.8. Statistical Analysis

All in vitro experiments were performed in at least triplicate. Data are presented as mean ± standard deviation (SD). Statistical significance between groups was determined by one-way analysis of variance (ANOVA) followed by Tukey’s post hoc test using GraphPad Prism software (version 9.5.1). A value of *p* < 0.05 was considered statistically significant.

### 3.9. Molecular Docking

The crystal structures of COX-2 (PDB ID: 5KIR) and PDE4B (PDB ID: 3WE4) were retrieved from the Protein Data Bank. The selection of these structures was based on their high resolution, completeness of the relevant domains (particularly the active site), and co-crystallization with a high-affinity inhibitor, ensuring the reliability of the binding site geometry for docking studies [72,73]. Protein preparation was performed using AutoDockTools (version 1.5.6) software. Water molecules and heteroatoms were removed, Kollman charges were added, and polar hydrogen atoms were merged. The 3D structure of compound **14** was drawn using Marvin JS (version 25.3.0) and energy-minimized using Avogadro (version 1.2.0) software with the MMFF94 force field. The ligand was prepared for docking by assigning Gasteiger charges and setting rotatable bonds in AutoDockTools (version 1.5.6) [74,75].

The grid boxes for docking were defined to encompass the known active sites of the proteins. For COX-2 (5KIR), the center coordinates were set to x = 15.8, y = 65.3, z = 40.4 with dimensions of 20 × 20 × 20 Å^3^, as referenced from the co-crystallized ligand. For PDE4B (3WE4), the center coordinates were set to x = 33.4, y = 33.0, z = 67.3 with dimensions of 20 × 20 × 20 Å^3^, based on the location of the native inhibitor [75,76]. Molecular docking was performed using AutoDock Vina 1.2.5. The exhaustiveness parameter was set to 400 to ensure a comprehensive search. For each ligand, 10 conformations were generated, and the pose with the most favorable (most negative) binding affinity (ΔG, kcal/mol) was selected for further analysis. The visualization of protein-ligand interactions and the creation of figures were conducted using Discovery Studio Visualizer (version 21.1.0.20298) [76,77].

### 3.10. Molecular Dynamics

MD simulations were performed using GROMACS 2023.3 software with the all-atom force field AMBER99SB-ILDN [78]. MD simulations were conducted for a system comprising two proteins under study, along with the docked poses of compound **14** and two positive control compounds. The pdb2gmx tool was employed to generate topology files for the enzyme binding structures, and the TIP3P water model was selected [78,79]. AmberTools 23 was utilized to create topology files for the studied ligands [80]. The protein-ligand complexes were placed in a solvent box using the solvate tool, and the genion tool was used to neutralize the charges by adding counter ions into the water box [81,82]. Energy minimization was performed for all systems using the steepest descent algorithm. Subsequently, NVT and NPT equilibration were carried out for the systems using V-rescale and Berendsen algorithms to achieve a temperature of 300 K and a pressure of 1 bar, respectively. The mdrun tool was employed to perform MD simulations for 100 ns for all systems. Root mean square deviation (RMSD) and radius of gyration (Rg) calculations were outputted using the GROMACS program and visualized using XMGRACE software v 5.1.25 [82,83].

## 4. Conclusions

This study successfully isolated and identified fourteen compounds from the ethyl acetate extract of *P. pentandra*, expanding the known phytochemical profile of this medicinal plant. Among them, compound **14** was identified as the most promising anti-inflammatory agent. It demonstrated significant efficacy in vitro, effectively inhibiting NO production and suppressing the release of the pro-inflammatory cytokines TNF-α and IL-6 in LPS-stimulated RAW 264.7 macrophages. A key mechanistic insight was its inhibition of the NF-*κ*B signaling pathway, as evidenced by the prevention of p65 subunit nuclear translocation.

The integrated in silico approaches provided crucial complementary insights into its activity. Molecular docking revealed that compound **14** interacts with the key inflammatory targets COX-2 and PDE4B, showing a particularly strong predicted affinity for PDE4B. Subsequent MD simulations confirmed the formation of stable complexes with both enzymes, with superior stability observed in the PDE4B complex.

In summary, our multi-faceted investigation provides a strong scientific foundation for the traditional use of *P. pentandra* in treating inflammatory conditions. We identify compound **14** as a novel, potent natural lead compound with a multi-mechanistic anti-inflammatory profile, encompassing the inhibition of effector enzymes (COX-2/PDE4), pro-inflammatory mediators (NO), cytokines (TNF-α, IL-6), and the central NF-*κ*B transcriptional pathway. These findings not only contribute to the understanding of the anti-inflammatory mechanisms of *P. pentandra* constituents but also highlight the value of this plant as a source for developing new natural anti-inflammatory agents or functional ingredients. Future work should focus on further mechanistic studies, in vivo efficacy in other inflammatory models, and optimization of this compound for potential therapeutic applications.

## Figures and Tables

**Table 1:** Docking results of compound **14** to the target proteins COX-2 and PDE4B.

Compound	PDB ID	Binding Affinity (kcal/mol)	Hydrogen Bond	Hydrophobic Interaction
**14**	5KIR	−6.138	Asn350	Lys358, Asp347, His356, Ser581, Ser579, Gly354
	3W5E	−9.361	Leu502, Glu509	Glu304, Met347, Phe414, Glu509
rofecoxib	5KIR	−8.9	Arg513	Leu352, Val349, Val523, Ser353, Ala527, His90
NVW	3W5E	−12.05	Gln443	Ile410, Met503, Leu502, Phe414, Met347

## Data Availability

The original contributions presented in this study are included in the article. Further inquiries can be directed to the corresponding author.
